# Simul-seq: combined DNA and RNA sequencing for whole-genome and transcriptome profiling

**DOI:** 10.1038/nmeth.4028

**Published:** 2016-10-10

**Authors:** Jason A Reuter, Damek V Spacek, Reetesh K Pai, Michael P Snyder

**Affiliations:** 1 Department of Genetics, Stanford University School of Medicine, Stanford, California, USA; 2 Department of Pathology, Stanford University School of Medicine, Stanford, California, USA

## Abstract

Paired DNA and RNA profiling is increasingly employed in genomics research to uncover molecular mechanisms of disease and to explore personal genotype and phenotype correlations. here, we introduce Simul-seq, a technique for the production of high-quality whole-genome and transcriptome sequencing libraries from small quantities of cells or tissues. We apply the method to laser-capture-microdissected esophageal adenocarcinoma tissue, revealing a highly aneuploid tumor genome with extensive blocks of increased homozygosity and corresponding increases in allele-specific expression. Among this widespread allele-specific expression, we identify germline polymorphisms that are associated with response to cancer therapies. We further leverage this integrative data to uncover expressed mutations in several known cancer genes as well as a recurrent mutation in the motor domain of *KIF3B* that significantly affects kinesin–microtubule interactions. Simul-seq provides a new streamlined approach for generating comprehensive genome and transcriptome profiles from limited quantities of clinically relevant samples.

Integration of both DNA and RNA sequencing data enables a variety of analyses that are useful for exploring the genetics of normal phenotypic variation and disease. In addition to enumerating global patterns of gene expression, RNA sequencing data provides an orthogonal verification of DNA variant calls and can be used to prioritize expressed candidates, which are more likely to exert biologic effects. In cancer, for example, roughly a third of the somatic single-nucleotide variants (SNVs) that fall within coding regions can also be observed in the RNA^1^, providing a biologic filter for candidate driver mutations. Furthermore, combined DNA and RNA profiling is useful for characterizing regulatory variation^2–4^, RNA editing^5^ and allele-specific expression^6–8^, important contributors to phenotypic diversity and disease.

Currently, most integrative experiments are performed in parallel and on distinct cell populations, a strategy that requires lengthy library preparation times and potentially exacerbates variability on account of sample heterogeneity. Single-cell integrative sequencing approaches, genome and transcriptome sequencing (G&T-seq)^9^ and gDNA and mRNA sequencing (DR-seq)^10^, have recently produced the first genome-wide glimpses of the correlation between copy number and expression at a cellular level. However, due to the large technical variance and coverage gaps inherent in current single-cell sequencing approaches, these new methods have limited utility in contexts where more comprehensive genomes and transcriptomes are required. Moreover, both methods still require the DNA and RNA libraries to be generated independently.

Our simultaneous DNA and RNA sequencing method, Simul-seq, leverages the enzymatic specificities of the Tn5 transposase and RNA ligase to produce whole-genome and transcriptome libraries without physical separation of the nucleic acid species (Fig. 1a), reducing the library preparation time compared with that of standard independent library approaches (Supplementary Fig. 1a). Simul-seq also employs a ribosomal depletion step, thereby maintaining many biologically relevant classes of noncoding RNAs. Additionally, Simul-seq incorporates dual 5′ and 3′ indices specific for both DNA and RNA molecules, minimizing cross contamination caused by spurious ligation and tagmentation or by template switching during pooled PCR. Finally, differential amplification from distinct RNA and DNA adapter sequences can be used to adjust the read outputs derived from either library.

## Results

### Simul-seq efficiently produces distinct RNA-seq and DNA-seq data

**Figure 1:**
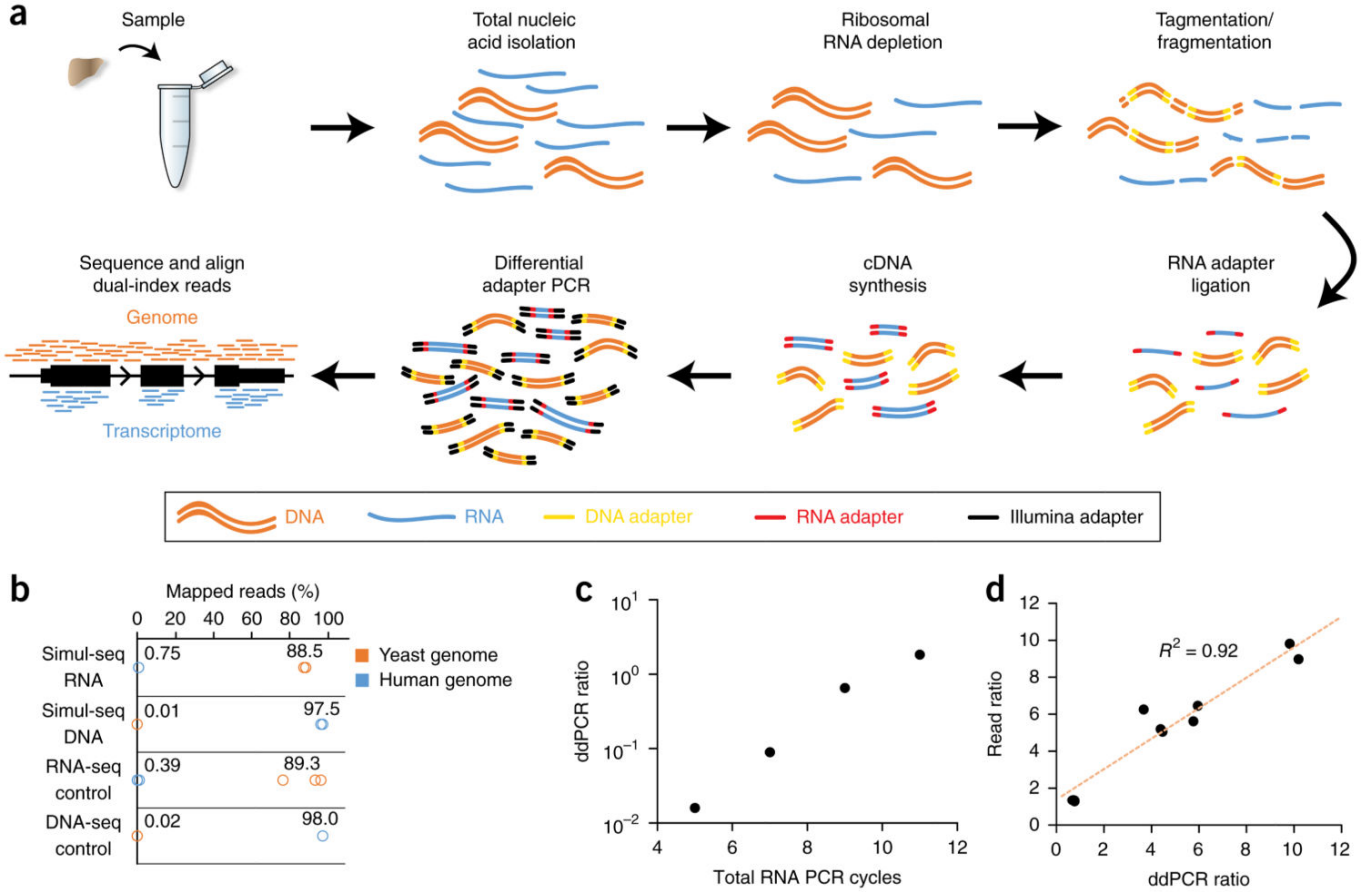
Simultaneous, single-tube sequencing of DNA and RNA. (**a**) Schematic of Simul-seq method. (**b**) Cross-species mapping rates for Simul-seq libraries produced from a mixture of yeast mRNA and human genomic DNA (*n* = 2) as well as yeast RNA-seq (*n* = 3) and human DNA-seq controls (*n* = 2). (**c**) Droplet digital PCR (ddPCR) assays on Simul-seq libraries (*n* = 3 technical replicates per library) with varying amounts of RNA-specific PCR amplification followed by an additional five cycles of PCR with primer sets for both RNA and DNA. (**d**) DNA and RNA library ratios measured by ddPCR (*n* = 3 technical replicates per library) are correlated with subsequent read ratios.

To rigorously assess the specificity of the Simul-seq method, we first produced libraries derived from a mixture of 50 ng of human genomic DNA and 100 ng of yeast mRNA (Supplementary Fig. 1b). We quantified the presence of both DNA-seq and RNA-seq libraries in the pool using droplet digital PCR (ddPCR; Supplementary Fig. 1c,d). Subsequent sequencing and alignment of the dual-indexed reads to the yeast and human genomes revealed cross-species mapping rates that were similar to those observed in yeast RNA-seq and human DNA-seq libraries produced independently (Fig. 1b), indicating that the Simul-seq method specifically barcodes the DNA and RNA with distinct adapters. Next, we leveraged these adapters to optimize read outputs for various applications and starting material inputs using differential PCR. To verify this approach, we varied the number of PCR cycles with RNA primers alone while holding the number of cycles with both DNA and RNA primers constant. Inclusion of RNA-specific cycles increased the fraction of the total library derived from RNA, as measured by ddPCR (Fig. 1c). Moreover, ddPCR quantification of the DNA and RNA constituents before sequencing was also highly correlated with subsequent read outputs (Fig. 1d), enabling users to perform quality control on the mixed libraries before high-throughput sequencing.

### Simul-seq DNA sequencing data is of high quality

**Figure 2:**
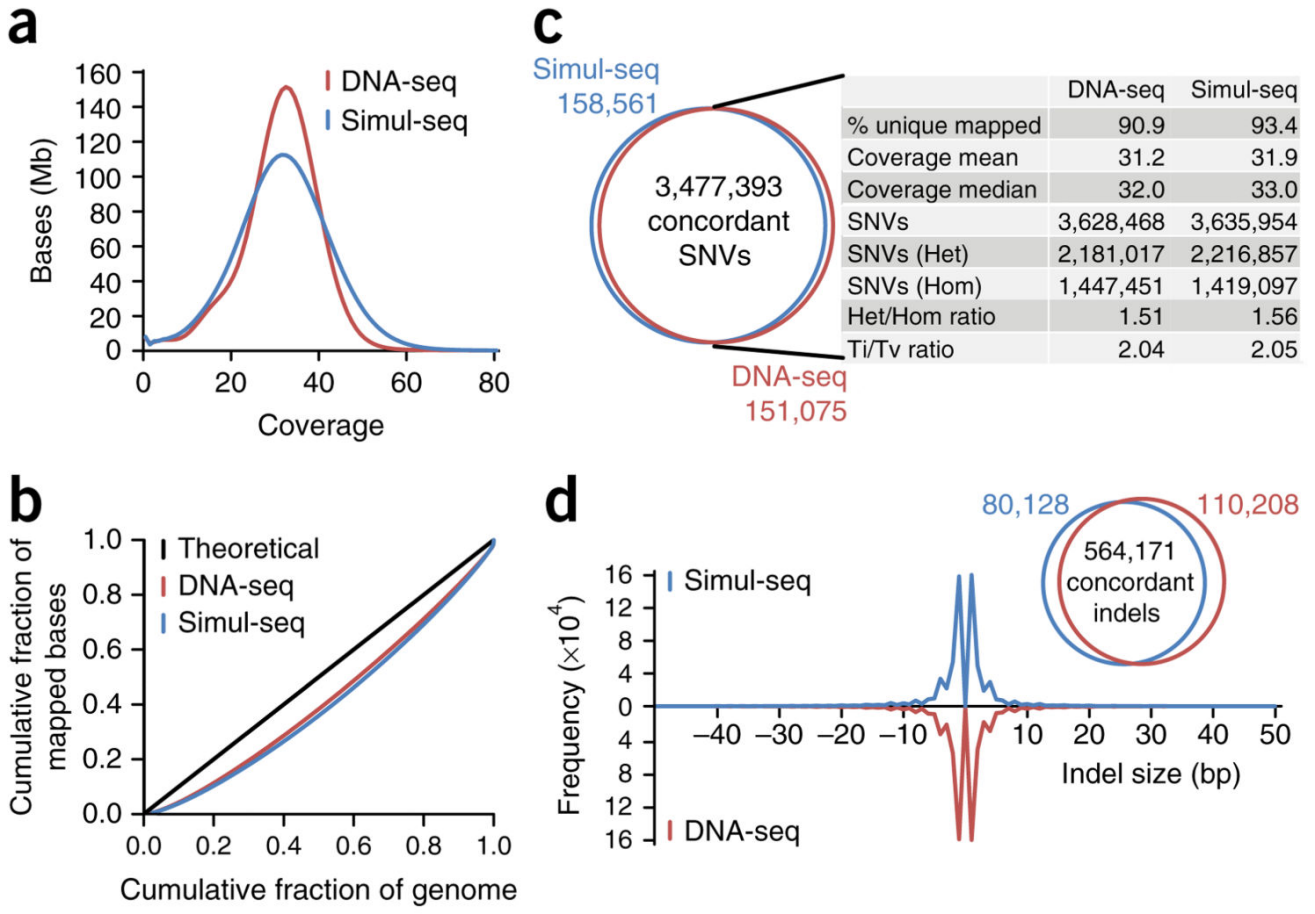
Characterization of Simul-seq whole-genome data. (**a**) Coverage distributions for Simul-seq and DNA-seq genomes of the same individual^11^. (**b**) Lorenz curves for the cumulative fraction of the covered genome versus the cumulative fraction of total mapped bases. Black line indicates the theoretical limit for independent sampling. (**c**) Comparison of single-nucleotide variant (SNV) calls between Simul-seq and DNA-seq genomes. (**d**) Comparison of insertion and deletions (indels) calls and size distributions between Simul-seq and DNA-seq genomes.

To benchmark Simul-seq against established library preparation methods, we next applied the approach to fibroblasts derived from an individual who had previously been subjected to whole-genome sequencing^11^. In parallel, we also prepared independent RNA-seq libraries from these cells using an analogous RNA-ligase-based protocol. For the Simul-seq library, we obtained 560,218,621 and 57,091,162 dual-indexed DNA and RNA 101-bp paired-end reads, respectively (Supplementary Table 1). 93% of Simul-seq DNA reads mapped to the genome, producing an average genomic depth of 31.9 × (Fig. 2a). Although the Simul-seq coverage distribution was consistent with the distribution obtained from a library previously generated using an established DNA-seq method^11^ (Fig. 2a), the distribution exhibited some sequencing bias characteristic of the Tn5 transposase^12^. To further explore potential coverage biases, we generated Lorenz curves comparing the cumulative fraction of mapped bases with the cumulative fraction of the genome covered. Both the Simul-seq and the DNA-seq control genomes exhibited comparable read distributions (Fig. 2b), indicating that pooled DNA and RNA library preparation and sequencing does not introduce sequencing bias in excess of standard methods.

Whole-genome sequencing is generally performed to identify variants that are polymorphic among populations or associated with disease. Therefore, we next compared variant calls between the Simul-seq and control DNA-seq genomes. Of the 3,635,954 SNVs determined in the Simul-seq genome, 95.6% were concordant with SNVs called in the standard DNA-seq genome (Fig. 2c). In addition, the identity and size distribution of small insertions and deletions (indels) identified in the Simul-seq genome were similar to those obtained from the DNA-seq genome, with 87.5% of Simul-seq-derived indels exhibiting concordance with the standard genome (Fig. 2d). These degrees of concordance were comparable to those observed from previously published biologic replicates using a standard DNA-seq approach^11^ (Supplementary Fig. 2a,b), demonstrating that Simul-seq produces high-quality whole-genome data.

### Simul-seq RNA sequencing data is of high quality

**Figure 3:**
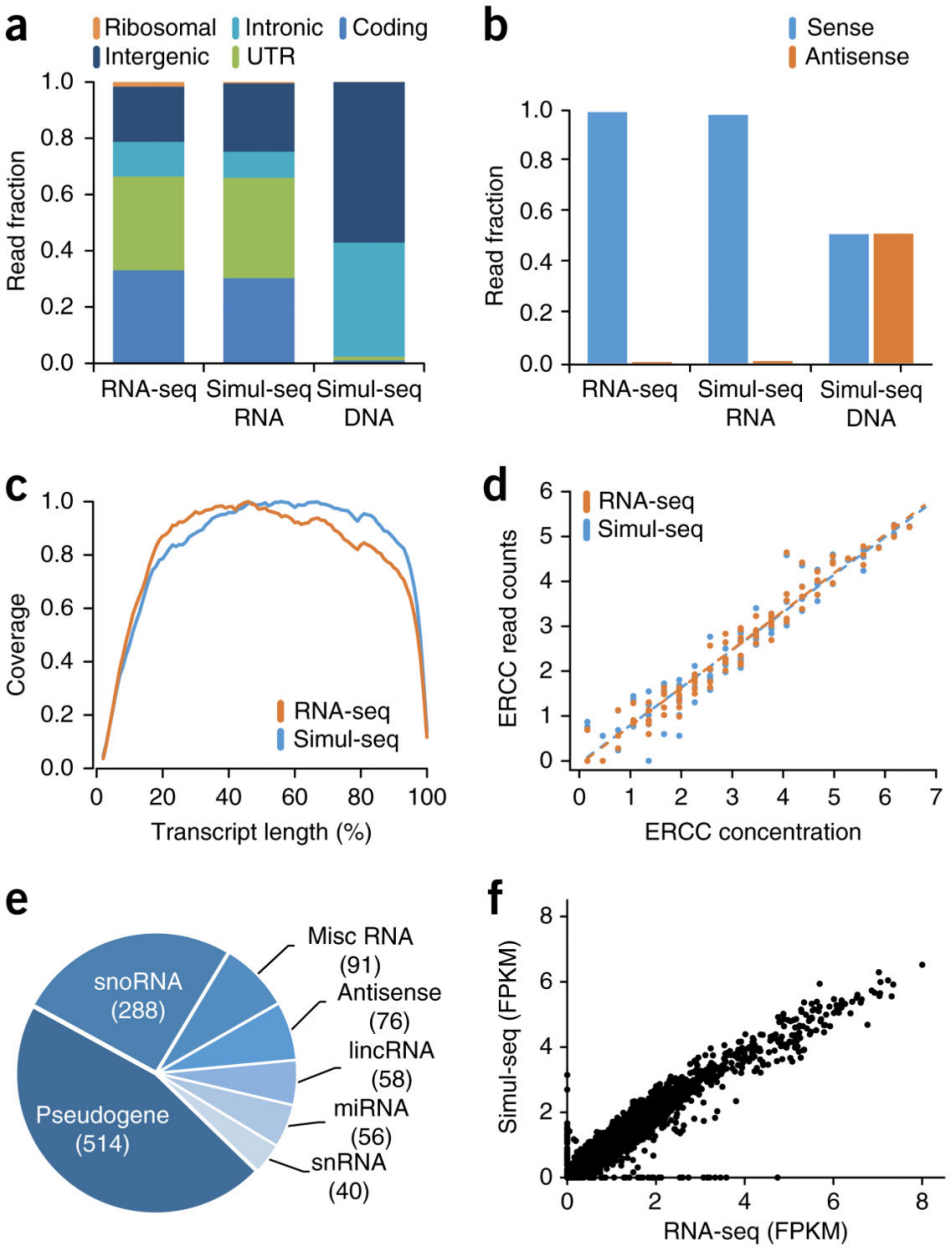
Characterization of Simul-seq transcriptome data. (**a,b**) Genomic distribution and strand specificity of Simul-seq RNA-indexed reads compared to RNA-seq control. Simul-seq DNA-indexed reads are included as a control. (**c**) Distribution of normalized transcript coverage for Simul-seq and RNA-seq transcriptome data. (**d**) Correlation between External RNA Controls Consortium (ERCC) spike-in control log_10_ RNA concentrations versus the average log_10_(RPKM) for Simul-seq (Spearman's ρ = 0.97) and RNA-seq (Spearman's ρ = 0.98) replicates (*n* = 2). Note, RPKM values of 0 have been shifted to 1, and all ERCC transcripts are shown. (**e**) Pie chart of Ensembl genes (FPKM ≥ 5) with noncoding biotypes from the Simul-seq transcriptome. Misc RNA, miscellaneous RNA; lincRNA, long intergenic noncoding RNA; miRNA, microRNA; snoRNA, small nuceolar RNA; snRNA, small nuclear RNA. (**f**) Scatter plot of log_10_(FPKM + 1) values across all genes measured in the Simul-seq or RNA-seq data sets (Spearman's ρ = 0.97).

Next, we examined the quality of the RNA sequencing data. Similar to RNA-seq control data, Simul-seq RNA reads were effectively depleted for ribosomal sequences and mapped primarily to transcribed regions of the genome (Fig. 3a). Simul-seq RNA reads were also highly strand specific and evenly distributed across the length of transcripts (Fig. 3b,c), enabling accurate transcriptome quantification and isoform analysis. As a control, Simul-seq DNA reads mapped primarily to intronic and intergenic regions of the genome and were evenly distributed between each DNA strand, as expected (Fig. 3a,b). To rigorously assess the technical variation of transcript quantification, External RNA Controls Consortium (ERCC) RNA standards^13^ were spiked into the total nucleic acid mixture. Simul-seq produced ERCC transcript measurements that were both highly correlated with the known ERCC concentrations as well as with RNA-seq control ERCC measurements (Fig. 3d). The Simul-seq-derived transcriptome contained 7,992 protein-coding genes as well as an additional 1,123 noncoding genes that would be largely undetected with poly-A enrichment (Fig. 3e and Supplementary Fig. 3). Moreover, fragments per kilobase of transcript per million fragments mapped (FPKM) measurements were both reproducible and well correlated with RNA-seq control FPKMs (Fig. 3f and Supplementary Fig. 4). Taken together, these experiments demonstrate that the Simul-seq protocol efficiently produces high-quality whole-genome sequencing data and RNA sequencing data, allowing for the comprehensive profiling of genomic and transcriptomic variation from the same cell population. In addition, we have applied the method to as few as 50,000 fibroblasts, obtaining coverage distributions and variant calls (Supplementary Fig. 5a,b) as well as FPKM and ERCC expression data (Supplementary Fig. 5c,d) that were both reproducible and well correlated with our previous results.

### Application of Simul-seq to cancer

**Figure 4:**
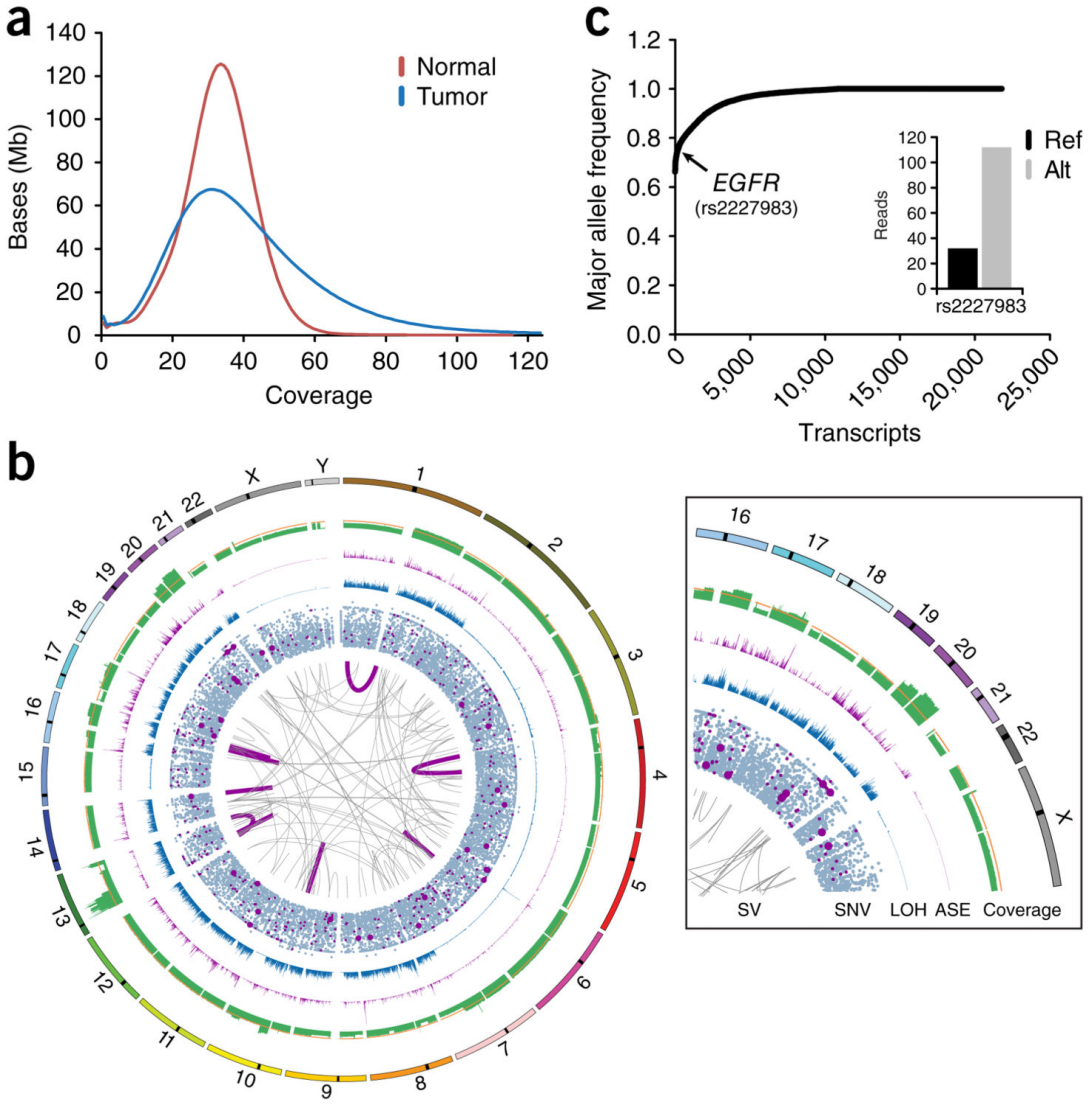
Comprehensive genome and transcriptome profiling of esophageal adenocarcinoma (EAC). (**a**) Coverage distributions for Simul-seq tumor genome and DNA-seq normal genomes. (**b**) Circos plot of somatic events in the tumor genome. The innermost ring depicts large structural variants (SVs), with expressed gene fusions highlighted in dark magenta. The second ring is a scatter plot of somatic single-nucleotide variants (SNVs), where an increased radial distance represents an increasing variant allele quality in the tumor genome. Dark magenta data points indicate expressed somatic SNVs, with the radius of expressed, nonsynonymous somatic mutations enlarged. The third ring is a histogram of the total number of heterozygous positions in the normal that are called homozygous in the tumor (LOH) per 100 kb. The fourth ring is a histogram of the number of transcripts exhibiting allele-specific expression (ASE) per 100 kb. The fifth ring corresponds to the normalized average coverage over 100-kb bins, whereas the orange line indicates the genome-wide average coverage. The outermost ring represents chromosome annotations. (**c**) Scatter plot for the average major allele frequencies for each transcript exhibiting allele-specific expression. Inset depicts reference (ref) and alterative (alt) RNA read counts for a known *EGFR* polymorphism.

Integrative DNA and RNA profiling is increasingly employed in cancer genomics to distinguish driver mutations of various types (e.g., protein coding, regulatory, structural variants, etc.) from the multitude of passenger mutations^1,14,15^. To test Simul-seq in this tissue context, we applied the method to laser-capture-microdissected material (∼150 μg) isolated from a male subject with metastatic esophageal adenocarcinoma (EAC). Deep sequencing of the Simul-seq EAC library produced 727,341,682 DNA and 191,398,961 RNA 101-bp dual-indexed paired-end reads, with 95.1% and 79.4% of the reads mapping to the genome and transcriptome, respectively (Supplementary Table 1). Similarly to the data acquired from fibroblasts, the Simul-seq RNA reads primarily mapped to transcribed regions, were highly strand specific and evenly distributed over transcripts (Supplementary Fig. 6a,b). However, the percentage of reads mapping to introns was increased for this library, suggesting an increased rate of intron retention and/or number of unspliced transcripts in this tumor specimen (Supplementary Fig. 6c). The tumor genome was sequenced to an average coverage of 38× and displayed a skewed coverage distribution indicative of large-scale copy-number alterations (Fig. 4a).

Comparing the Simul-seq tumor genome with a DNA-seq paired normal genome revealed a highly aneuploid genomic landscape, with somatic evidence for 142 structural variants and 9 expressed gene fusions as well as 15,607 SNVs and 2,904 indels (Fig. 4b and Supplementary Tables 2–5). Globally, the ratio of heterozygous to homozygous SNPs for the tumor genome was 0.49, an exceptional deviation from the typically observed ratio of ∼1.5 (Fig. 2c) that indicated widespread loss of heterozygosity (LOH) (Fig. 4c). Analysis of allele-specific expression using the Simul-seq EAC transcriptome data provided further support for extensive LOH, with 92.9% of the identified allele-specific transcripts exhibiting average major allele frequencies of greater than or equal to 0.9 (Fig. 4c and Supplementary Table 6). Given the high levels of LOH-induced allele-specific expression (ASE) in the tumor, we hypothesized that damaging germline variants in tumor suppressor genes might be specifically expressed in the tumor. Indeed, we identified eight nonsynonymous variants in tumor suppressor genes (as defined by the TSGene 2.0 database^16^) where a PolyPhen-2 (ref. 17)- and SIFT^18^-predicted damaging allele was predominantly expressed (Supplementary Table 7).

To distill the 15,607 somatic SNVs into potential oncogenic mutations, we integrated the Simul-seq DNA and RNA data to identify 29 expressed nonsynonymous somatic mutations (Table 1 and Supplementary Table 4). In addition to representing potential driver mutations, these expressed protein-altering mutations are also possible neoantigens from which patient-specific immunotherapies may be derived^19–21^. Notably, three Cosmic Cancer census genes^22^ (*TP53, ATM* and *ESWR1*) were found to harbor expressed somatic missense mutations. While ESWR1 is typically a constituent of an oncogenic fusion protein, and the R45W mutation in the ATM serine/threonine kinase tumor suppressor is not yet characterized, the Y220C mutation is a known TP53 hotspot that decreases protein stability^23,24^. Moreover, we found that the *TP53* locus exclusively expressed the damaging allele (Table 1), exacerbating the loss of TP53 function and likely underpinning the widespread genomic instability observed in this tumor specimen. Interestingly, this patient also exhibited ASE for common germline polymorphisms in the epidermal growth factor receptor gene (*EGFR,* rs2227983) as well as the cyclin D1 gene *(CCND1,* rs9344) (Supplementary Table 6), polymorphisms that are associated with response to chemotherapeutic treatments^25–28^.

### Characterization of a recurrent mutation in a kinesin family gene

**Figure 5:**
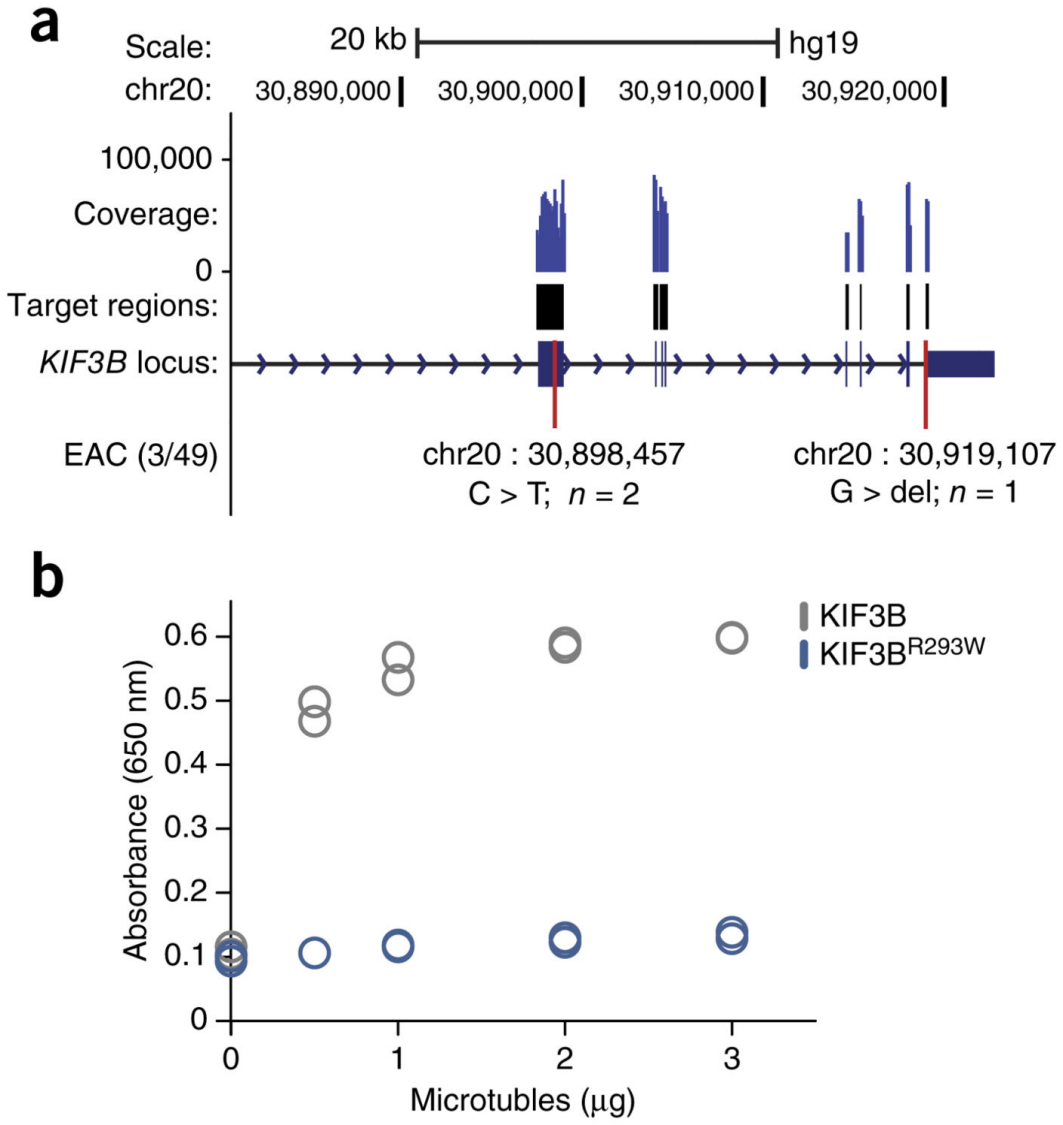
Identification and biochemical characterization of a recurrent mutation in KIF3B. (a) Schematic of *KIF3B* locus, including positions of mutations found in targeted resequencing of 49 esophageal adenocarcinoma patients and 25 paired controls. Coverage for representative sample is shown. Note, the original sample that was subjected to the Simul-seq protocol was included as a positive control. (b) Strip plot of ATPase activity (*n* = 2) for recombinant wild-type and R293W mutant KIF3B motor domains when incubated with increasing quantities of microtubules. Activity was quantitated using an endpoint measurement of free phosphate.

In addition to discovering clinically relevant alterations in known cancer genes, we observed an expressed arginine-to-tryptophan mutation in KIF3B (R293W), a type II kinesin motor protein. Although several kinesin family members have established roles in cancer^29^, *KIF3B* somatic coding mutations have not been previously described. KIF3B has been linked to the intracellular trafficking of several tumor suppressor genes^29,30^, and biochemical data have shown that substitution of specific arginine and lysine residues within the kinesin motor domain negatively impacts kinesin-microtubule association^31^. To further explore *KIF3B* mutation frequency in EAC, we performed targeted resequencing of the *KIF3B* locus in a cohort of 49 EAC samples, with 25 paired normals. Overall, *KIF3B* harbored verified nonsynonymous mutations in ∼6% of the tumor samples, and the R293W mutation was observed in a second independent patient (Fig. 5a and Supplementary Fig. 7a,b). To investigate the functional consequences of this recurrent R293W mutation, we purified recombinant wild-type and mutant KIF3B motor domains (Supplementary Fig. 8a,b). When compared with the wild-type domain, the mutant motor domain displayed a significantly reduced rate of ATP hydrolysis upon incubation with various concentrations of microtubules, suggesting that the R293W mutation abrogates kinesin–microtubule binding (Fig. 5b). Together, these results demonstrate the benefits of Simul-seq in providing comprehensive DNA and RNA data sets, leading to the annotation of several clinically important variants as well as the description of a functionally significant recurrent mutation.

## Discussion

As sequencing technologies advance and more individuals are profiled in both clinical and research settings, straightforward methods for generating comprehensive and accurate whole-genome and transcriptome sequencing data will become increasingly valuable. The combined sequencing of both DNA and RNA from single cells was recently enabled by the development of two methods, DR-seq^32^ and G&T-seq^33^. Simul-seq provides a complementary approach that focuses on producing comprehensive DNA and RNA profiles from limited quantities of tissues or cells rather than single cells. In contrast to previous dual-sequencing approaches, Simul-seq generates a single pooled library, and thus both reduces the library preparation time and keeps paired data sets physically linked. Importantly, whereas DR-seq and G&T-seq depend upon polyadenylation to distinguish RNA transcripts from genomic DNA, the use of RNA ligase in Simul-seq allows for a ribosomal RNA depletion step. Therefore, Simul-seq retains biologically and clinically important nonpolyadenylated RNA transcripts and may reduce 3′ bias for samples with lower RNA quality^34,35^. Overall, Simul-seq produces high-quality DNA and RNA sequencing data, enabling genotype and phenotype comparisons in a single workflow.

Cancer genome interpretation is one scenario where integration of precise and comprehensive DNA and RNA landscapes has proven useful but can be challenging on account of limited starting material. Moreover, tumor heterogeneity increases the likelihood of discrepancies between genome and transcriptome profiles prepared in parallel on separate cell populations. Applying Simul-seq to laser-capture-microdissected tumor tissue revealed a highly aneuploid somatic landscape, including a recurrent R293W mutation in KIF3B that dramatically reduced kinesin–microtubule interaction. Although the ∼6% mutation frequency that we observed is consistent with recently published data from whole-genome sequencing of 22 esophageal adenocarcinomas^36^, *KIF3B* has not been classified as a cancer gene in large-scale EAC exome sequencing studies^37,38^. These efforts, however, are still largely statistically underpowered^14^. Intriguingly, overexpression of C-terminal truncations of KIF3B-induced aneuploidy in NIH3T3 cells^39^. Moreover, KIF3B has been linked to the intracellular trafficking of several tumor suppressors, including the adenomatous polyposis coli (APC)^30^ and von Hippel–Lindau (VHL)^29^ proteins. Together, our findings suggest that additional experiments are warranted to delineate specific functional roles for KIF3B mutation in esophageal tumorigenesis.

In addition to the novel KIF3B mutation, we also identified a number of clinically relevant variants in this EAC patient sample. We observed a known TP53 hotspot mutation (Y220C) that destabilizes the TP53 protein at body temperatures^24^ and is also a target of several small molecules designed to restore TP53 function in tumors^23,40^. TP53 inactivation followed by whole-genome duplication and chromosomal catastrophe is a frequent trajectory for EAC development^36,41^ and is consistent with our observations for this tumor. Among the widespread LOH induced by this genomic instability, we detected ASE for germline variants with pharmacogenomic links to the efficacy of cancer therapies used in EAC. The *EGFR* polymorphism (rs2227983) observed in this patient is associated with increased survival of colorectal cancer patients treated with Cetuximab^27,28^, perhaps via attenuation of EGFR pathway signaling^42^. In contrast, the patient harbored a second variant in *CCND1* (rs9344) that is inversely correlated with overall survival in colorectal cancer patients treated with Cetuximab^43^. In both cases, however, the beneficial allele was predominantly expressed in the tumor, suggesting a positive overall response. Taken together, our results in this EAC patient both highlight the utility of Simul-seq as well as the many benefits of acquiring combined DNA and RNA profiles for genome interpretation and personalized medicine.

## Online Methods

### Sample acquisition

The male-patient-derived fibroblasts used in this study were collected and derived with informed patient consent under a protocol approved by the Institutional Review Board at Stanford University Medical Center (IRB17576). Cells tested negative for mycoplasma and were cultured with DMEM supplemented with 10% fetal bovine serum (FBS). The deidentified male esophageal cancer sample was obtained from Stanford Cancer Institute's Tissue Repository and was exempt from IRB requirements by the Stanford Research Compliance Office. Investigators were not blinded to experimental groups, and no power calculation was performed before experiments to ensure detection of a prespecified effect size.

### DNA/RNA extraction

For the mixing experiments, yeast mRNA was obtained from Clontech (Clontech: 636312) and human genomic DNA was isolated using the DNA Mini kit (Qiagen: 51304). For all other Simul-seq experiments, total nucleic acids were extracted using the RNeasy Mini kit (Qiagen: 74104) per manufacturer's instructions, except the optional DNase I treatment was not performed. DNA and RNA were then quantified using the Qubit DNA HS and RNA HS (Thermo Fisher: Q32851, Q32852), respectively. For fibroblast experiments, extraction began with 1 × 10^6^ cells, whereas the laser-capture-microdissected (LCM) tumor library started with approximately 150 μg of tissue (based on isolating ∼150 × 10^6^ μm^3^ and assuming an average tissue density of 1.0 g/cm^3^). The quality of the starting total RNA was measured using Bioanalyzer, with RNA integrity number (RIN) values ranging from 8 for LCM-isolated tissue to 10 for LCM-isolated cells. For Simul-seq library preparations, ERCC spike in mixture A (Life Technologies: 4456740) was added per manufacturer's instructions before the ribosomal RNA depletion step.

### Ribosomal depletion

Ribosomal RNA sequences were depleted from the total nucleic acid mixture using Ribo-Zero gold (Illumina: MRZG126) and following the manufacturer's instructions. To reduce potential hybridization to genomic DNA sequences; however, the standard 70 °C hybridization step was changed to 65 °C. Ribosomal RNA depletion began with the recommended amount of total RNA (1 μg for LCM tissue to 5 μg for fibroblasts). For 50,000 fibroblast experiments, ∼400 ng of total RNA was used. Following ribosomal RNA depletion, the total nucleic acid mixture was purified using RNA Clean and Concentrator 5 columns (Zymo Research: R1015) and quantified using high-sensitivity DNA and RNA Qubit reagents as above.

### Simul-seq protocol

Unless otherwise noted, reagents were from New England Biosciences (NEB: E7330S) or Illumina (Illumina: FC-121-1031). Simultaneous RNA fragmentation and DNA tagmentation was achieved by mixing 25 μl of TD buffer, 5 μl of TDE, 1 μl RNase III (0.5 U, NEB: E6146S) and 19 μl of DNA/RNA consisting of 30-50 ng of genomic DNA and 10–100 ng of ribodepleted RNA. This reaction was incubated for 5 min at 55 °C, and the thermocycler was cooled to 10 °C before the reaction was placed on ice. 100 μl Ampure XP RNAclean beads (Beckman Coulter: A63987), or 2× the reaction volume, were then added to the reaction and incubated for 10–15 min to bind the nucleic acids. The beads were placed on a magnet stand until clear, washed twice with 400 μl of 80% ethanol and dried for 10 min at room temperature The total nucleic acids were eluted from the dried beads using 7 μl of H_2_O. To remove secondary RNA structure, 6 μl of the eluate and 1 μl of the 3′ ligation adapter were first heated to 65 °C for 5 min and then immediately placed on ice. For ligation of the 3′ adapter to the RNA molecules, 10 μl of 3′ ligation buffer and 3 μl of 3′ ligation enzyme mix were added and incubated for 1 h at 25 °C in a thermal cycler with the lid heated to 50 °C. To reduce adapter–adapter ligation products, 1 μl of the reverse transcription primer (SR RT primer) and 4.5 μl of H_2_O were added to the 3′ adapter ligation reaction and incubated in a PCR machine for 5 min at 65 °C, 15 min at 37 °C, 15 min at 25 °C and held at 4 °C until the next step. To ligate the 5′ adapter, 1 μl of 5′ SR adapter, which had been previously heated to 70 °C and then placed on ice, along with 1 μl of 5′ ligation buffer and 2.5 μl of 5′ ligase enzyme mix were added to the 3′ adapter-ligated and SR-RT-primer-hybridized RNA. This reaction was incubated for 1 h at 25 °C with the lid heated to 50 °C and then placed on ice. First-strand cDNA synthesis was performed by adding 8 μl of first-strand reaction buffer, 1 μl of murine RNase inhibitor and 1μl of ProtoScript II reverse transcriptase to the previous mixture and incubating the reaction for 1 h at 42 °C with the lid heated to 50 °C. 48 μl of Ampure XP beads (Beckman Coulter: A63880), or 1.2× of the reaction volume, were then used to clean up the cDNA and transposed genomic DNA. The beads were incubated for 5–10 min with the DNA, washed twice with 80% ethanol and mixed with 26.5 μl of H_2_O to elute the DNA. PCR conditions varied depending on whether differential PCR was performed. DNA libraries were amplified using standard Nextera indexing primers. RNA libraries were amplified with a custom I5 indexing primer AATGATACGGCGACCACCGAGATCTA CACTATCCTCTGTTCAGAGTTCTACAGTCCG-s-A, where -s- indicates a phosphorothioate bond, and a standard I7 indexing primer. For differential PCR, 25.5 μl of the eluate was combined with 1.25 μl of each RNA indexing primer (10 mM stock) and 12 μl Nextera PCR Master Mix (NPM) and then thermocycled as follows: 72 °C for 3 min; 98 °C for 30 s; then two to seven cycles of 98 °C for 10 s, 62 °C for 30 s and 72 °C for 3 min; before a final hold at 4 °C. After this hold, the reaction was removed from the thermocycler and combined with 12.5 μl of a master mix comprising 2.5 μl of each DNA indexing PCR primer (5 mM stock), 5 μl of PPC and 5 μl NPM. This combined reaction was then subjected to five additional cycles using the same program described above. The fibroblast, LCM and 50,000 fibroblast Simul-seq libraries used two, four and seven cycles of RNA-specific PCR, respectively. The final libraries were cleaned using 66 μl Ampure XP beads as described above and eluted in 12 μl of H_2_0. To quality control the dual-indexed libraries, we performed high-sensitivity Qubit DNA and Bioanalyzer assays prior to sequencing of paired-end 101 bp reads on Illumina HiSeq or MiSeq machines. A typical Simul-seq library will be approximately 10 ng/ml, with an average size distribution of ∼350 bp (Supplementary Fig. 1b). A detailed description of Simul-seq reagents, equipment and a step-by-step protocol can be found in the Supplementary Note.

### Read processing and alignment

For both DNA and RNA reads, Cutadapt v1.8.1 (ref. 44) was used to trim the paired-end adapter sequences. Only trimmed reads longer than 30 bases and with a quality score >20 were aligned. For the DNA barcoded reads, 5′-CTGTCTCTTATACACATCTCCGAGCCCACGAGAC-3′ and 5′- CTGTCTCTTATACACATCTGACGCTGCCGACGA-3′ sequences were used to trim the adapter sequences. For RNA bar-coded reads, 5′-AGATCGGAAGAGCACACGTCTGAACTCCAG TCAC- 3′ and 5′-GATCGTCGGACTGTAGAACTCTGAACGTG TAGATC-3′ sequences were used to trim the adapter sequences.

DNA libraries were processed and analyzed using the Bina Technologies whole-genome analysis workflow with default settings. Briefly, libraries were mapped with BWA mem 0.7.5 software^45^ to hg19 and then realigned around indels with GATK IndelRealigner^46^. Next, base recalibration was performed with GATK BaseRecalibrator taking into account the read group, quality scores, cycle and context covariates. Variants were called with GATK HaplotypeCaller with the parameters–variant_index_ type LINEAR-variant_index_parameter 128000. VQSR was used to recalibrate the variants, first with GATK VariantRecalibrator and then ApplyRecalibration. For the cross-contamination analysis shown in Figure 1b, Simul-seq DNA-seq-indexed reads were mapped to hg19 and SacCer3 using Bowtie2 (ref. 47) with default settings.

RNA libraries were also processed and analyzed using Bina Technologies RNA analysis using default settings. Briefly, TopHat 2.0.11 (ref. 48) was used to map libraries to hg19, and Cufflinks^49^ was then used to perform per-sample gene expression analysis. Finally, Cuffdiff was used to find differential expression between replicates and different library types. For cross-contamination analysis shown in Figure 1b, Simul-seq RNA-indexed reads were mapped with TopHat to hg19 and SacCer3 using default settings.

### DNA and RNA QC analysis

Coverage plots were calculated from the Bina output. SNV and indel concordance between sequencing libraries was calculated using VCFtools v0.1.12 (ref. 50) on all variants annotated with a ‘passed’ filter. Summary statistics for SNVs were also calculated with VCFtools. Read fractions were calculated with Picard v1.92 (http://broadinstitute.github.io/picard) for the DNA and RNA sequencing libraries. Strand specificity and gene-body coverage were calculated with RSeQC 2.6.2 (ref. 51). For the analysis transcripts biotypes, the Simul-seq RNA data was mapped with TopHat using the Ensembl GENCODE annotations and quantitated with Cufflinks. Genes with FPKM values ≥5 were counted. Cuffdiff was used to compare log_10_(FPKM + 1) expression values between Simul-seq RNA libraries and control RNA-seq libraries.

#### Lorenz curves

Duplicates were removed from hg19-aligned reads using Picard v1.92, and Bedtools v2.18.0 (ref. 52) was used to calculate the coverage at every position in the genome. The file was then sorted by coverage, and cumulative sums for the fraction of the covered genome and the fraction of total mapped bases were calculated using custom scripts.

#### ERCC analysis

TopHat was used to align reads to ERCC reference using default settings. Next, duplicate reads were removed using Picard MarkDuplicates, and FeatureCounts^53^ was used to determine the total read counts for each ERRC transcript. Read counts were then normalized across transcripts and libraries using the RPKM methodology (i.e., reads per kb of transcript per million mapped reads). ERCC RPKM measurements for Simul-seq and RNA-seq replicates were averaged, zero values were set to one and then log_10_ transformed. ERCC transcript data for Simul-seq and RNA-seq replicates is shown (Supplementary Table 8).

### Droplet digital PCR

DNA:RNA ratios of between 5:1 to 10:1 are optimal for whole-genome and whole-transcriptome sequencing of human samples. ddPCR experiments were performed according to manufacturer's guidelines (Droplet Digital PCR Application Guide, Bulletin 6407 Rev A) using a Bio-Rad QX200 system. Briefly, custom qPCR assays were designed to the unique the DNA-seq and RNA-seq library adapter sequences and purchased from IDT as PrimeTime Std qPCR Assays (Supplementary Fig. 1c,d). These assays incorporated HPLC-purified probes with 5′ HEX or 6-FAM fluorophores and internal ZEN and 3′ Iowa Black FQ dual quenchers. 20 μl ddPCR reactions were assembled using diluted Simul-seq libraries (2 μl of a 10^−6^ dilution was typically sufficient but will vary depending on the starting library concentration). The ddPCR reactions were then subjected to the following cycling program: 10 min at 95 °C; 40 cycles of 30 s at 95 °C and 1 min at 60 °C, 10 min at 98 °C; and a hold at 4 °C. Triplicate reactions were done for each sample, and quantitation was performed using QuantaSoft version 1.3.2.

### Laser-capture microdissection

For LCM, 7 μm cryosections were placed onto 76 × 26 PEN glass slides (Leica: 11505158) and stored at −80 °C for up to 4 d. To guide the isolation process, serial sections were immunofluorescently stained with Keratin 8 (1:100; Abcam: ab668-100) and counterstained with Hoechst 33342 dye (2 mg/ml in PBS), marking the tumor epithelium and nuclei, respectively. On the day of laser capture, the LCM slides were stained with Cresyl violet according to the manufacturer's protocol (LCM staining kit, Ambion: AM1935). Immediately following staining, a Leica AS LMD system was used to isolate ∼150 × 10^6^ μm^3^ (or ∼150 μg) of esophageal adenocarcinoma tumor tissue. The LCM-isolated tissue was then subjected to the Simul-seq protocol; and 727,341,682 DNA and 191,398,961 RNA 101 bp paired-end reads were obtained using an Illumina HiSeq2000 machine. For all transcriptome analyses using Simul-seq RNA tumor data, 116,217,162 reads were analyzed.

### Somatic variant analysis

Somatic variant analysis was performed using Bina tumor-normal whole-genome calling workflow. Briefly, somatic variants with a Bina ONCOSCORE of greater than or equal to 5 were considered high confidence and reported. To identify somatic variants and generate the ONCOSCORE, Bina integrates JointSNVMix 0.7.5 (ref. 54), Mutect 2014.3-24-g7dfb931 (ref. 55), Somatic Indel Detector 2014.3-24-g7dfb931, Somatic Sniper 1.0.4 (ref. 56) and Varscan 2.3.7 (ref. 57) outputs. GATK ASEReadCounter was used to determine the variant and reference expression counts for somatic SNV positions in the tumor transcriptome data. The resultant somatic SNVs and indels are annotated in Supplementary Tables 4 and 5.

To determine large somatic structural variants (SVs), CREST^58^ was run on the tumor-normal paired genomic data. To refine the variant calls, we only reported SVs with greater than five supporting reads on both the 3′ and 5′ arms of the variant, which resulted in 142 total potential genomic SVs (Supplementary Table 2). Somatic SVs resulting in expressed gene fusions were independently determined using the INTEGRATE software package^59^, which incorporates tumor RNA sequencing data along with paired tumor-normal genome sequencing data. To refine this expressed fusion list, we only reported fusions with no evidence in the normal DNA data and at least one read of evidence for both the tumor DNA and RNA, which resulted in 9 potential expressed gene fusions (Supplementary Table 3). Circos software 0.63 (ref. 60) was used to display somatic variation in Figure 4b.

### Loss of heterozygosity

For the LOH analysis, heterozygous positions in the normal were selected in the VCF file using SNPsift^61^. GATK SelectVariants was then used to interrogate these heterozygous positions in the tumor VCF, classifying them as heterozygous or homozygous alternative. Heterozygous positions in the normal that were not present in the tumor VCF were considered homozygous reference and counted as LOH positions.

### Allele-specific expression

To examine LOH at the level of gene expression, allele-specific expression (ASE) in the tumor RNA was calculated for heterozygous positions called in the normal using ASEQ^62^. Briefly, GENOTYPE mode was run on a bam file derived from the paired normal genome with the following options: mbq = 20 mrq = 1 mdc = 5 htperc = 0.2. Next, ASE mode was run using a bam file from the tumor RNA with the following options: mbq = 20 mrq = 20 mdc = 10 pht = 0.01 pft = 0.01. This analysis was performed using an hg19 Ensembl transcript model and identified 21,797 transcripts—corresponding to 6,698 independent gene symbols—as exhibiting ASE (Supplementary Table 6). Circos was used to display the number of ASE transcripts in 100 kb bins in Figure 4b.

### Targeted resequencing of *KIF3B* locus

Overlapping primer sets were designed to capture all of the coding exons of the KIF3B locus (Supplementary Tables 9 and 10). Genomic DNA was isolated from 50 formalin-fixed paraffin embedded (FFPE) tumor samples as well as 26 paired normal samples using an AllPrep DNA/RNA FFPE kit (Qiagen: 80204) according to manufacturer's instruction. The original sample (02-28923-C9) that was subjected to the Simul-seq protocol was included as a positive control. The gDNA concentrations were normalized to 50 ng/μl and subjected to amplification on a Fluidigm Axess Array system, following manufacturer's recommendation (FC1 Cycler v1.0 User Guide rev A4). The resultant libraries were pooled, sequenced on a single HiSeq2000 lane and mapped using bowtie (see Supplementary Fig. 7a). SAMtools^63^ was used to generate a pileup, and SNVs were identified using four criteria: mapped to a targeted region, allele read fraction of ≥10%, mapping quality of ≥10 and coverage of ≥500. Using these criteria, three variants in *KIF3B* were identified and subsequently validated using pyrophosphate sequencing (see Supplementary Fig. 7b). A single tumor-normal pair (00-18224-A2) displayed a substantially higher number of variant calls yet a lower number of uniquely mapped reads, suggesting that these samples harbored increased rates of PCR errors induced by low-quality genomic DNA. Therefore, variants identified in these samples were not reported.

### Kinesin-microtubule interaction assays

Full-length kinesin proteins exhibit poor solubility in bacteria^64^. Therefore, wild-type and R293W mutant motor domains (amino acids 1–365) were amplified using the following primers: CATATGTCAAAGTTGAAAAGCTCAG and CTCGAGCTAGAGCCGAGCAAT CTCTTCCT. The PCR products were digested with NdeI/XhoI restriction enzymes and cloned into NdeI/XhoI-digested pET28a backbone, tagging the KIF3B motor domains on the N terminus. Recombinant KIF3B was purified using nickel affinity purification (Supplementary Fig. 8a,b). Briefly, bacterial pellets were lysed for 30 min on ice in lysis buffer (50 mM PIPES, pH 8.0, 1 mM MgCl_2_, 250 mM NaCl_2_, 250 μg/ml lysozyme, 250 mM ATP and protease inhibitors (Roche: 04693132001)). Lysates were pulse sonicated for three cycles of 18% amplitude (Bronson) for 5 s (0.5 s on and 1 s off), followed by 1 min on ice. Lysates were then cleared by centrifugation for 10 min at 4 °C and maximum speed. Cleared lysates were incubated with His-tag magnetic beads (Life Technologies: 10103D) for 1 h at 4 °C, washed 2× in washing buffer (50 mM PIPES, pH 8.0, 1 mM MgCl_2_, 250 mM NaCl_2_, 50 mM imidazole) supplemented with 250 mM ATP followed by an additional two washes in buffer excluding ATP. Beads were subsequently eluted in 25 mM PIPES, pH 8.0, 2 mM MgCl_2_, 125 mM NaCl_2_, and 250 mM imidazole. Kinesin ATPase end-point biochemical assays (Cytoskeleton: BK053) were performed in duplicate according to manufacturer's instructions with 0.4 μg of recombinant protein and increasing amounts of polymerized microtubules (see Fig. 5b).

## Supplementary Material

Supp Supplementary Table 1: Simul-seq and control library read counts and mapping rates.
Supplementary Table 2: Somatic SVs for Simul-seq EAC tumor genome
Supplementary Table 3: Somatic, expressed gene fusions in Simul-seq EAC tumor genome
Supplementary Table 4: VCF of somatic SNVs for Simul-seq EAC tumor genome
Supplementary Table 5: VCF of somatic indels for Simul-seq EAC tumor genome
Supplementary Table 6: EAC tumor ASE analysis at heterozygous SNV positions in the normal genome
Supplementary Table 7: ASE of annotated tumor supressor genes harboring damaging germline variants
Supplementary Table 8: Simul-seq RNA and RNA-seq ERCC spike-in transcript quantification
Supplementary Table 9: Genomic regions of KIF3B locus targeted for resequencing
Supplementary Table 10: Primer sets used in KIF3B targeted resequencing

## Figures and Tables

**Table 1:** Selected expressed somatic nonsynonymous variants in cancer-related genes

Gene	DNA (ref/alt)	RNA counts (ref/alt)	Protein	Cosmic census
*TP53*	T/C	0/76	Y220C	Yes
*ATM*	C/T	102/37	R45W	Yes
*EWSR1*	C/T	26/9	P122L	Yes
*KIF3B*	C/T	170/64	R293W	No
*MCM3AP*	G/A	5/127	R1207C	No
*FAT1*	C/T	11/44	V1274I	No
*MADD*	G/A	59/19	R225Q	No
*LRP1*	G/T	16/3	D2106Y	No
*H2AFY*	G/A	13/43	R4C	No
*ZNF615*	T/C	0/10	N154S	No
*CSTF1*	G/A	51/68	G26S	No
