# Supplementary material for: Simul-seq: combined DNA and RNA sequencing for whole-genome and transcriptome profiling
Source: Nat Methods. Author manuscript; Available in PMC 2017 Dec 18. (PMC5734913; doi:10.1038/nmeth.4028)
Supplement: Supp — lementary Table 1: Simul-seq and control library read counts and mapping rates. Supplementary Table 2: Somatic SVs for Simul-seq EAC tumor genome Supplementary Table 3: Somatic, expressed gene fusions in Simul-seq EAC tumor genome Supplementary Table 4: VCF of somatic SNVs for Simul-seq EAC tumor genome Supplementary Table 5: VCF of somatic indels for Simul-seq EAC tumor genome Supplementary Table 6: EAC tumor ASE analysis at heterozygous SNV positions in the normal genome Supplementary Table 7: ASE of annotated tumor supressor genes harboring damaging germline variants Supplementary Table 8: Simul-seq RNA and RNA-seq ERCC spike-in transcript quantification Supplementary Table 9: Genomic regions of KIF3B locus targeted for resequencing Supplementary Table 10: Primer sets used in KIF3B targeted resequencing [file NIHMS899760-supplement-Supp.pdf]

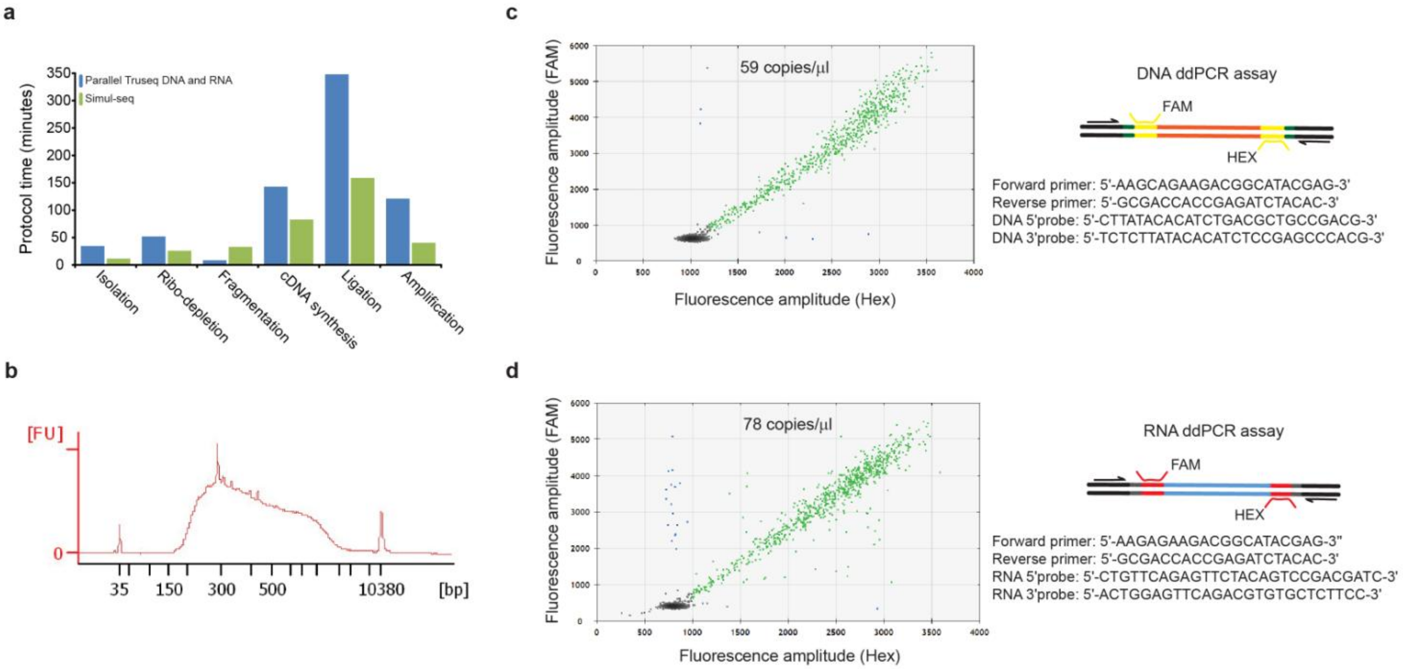

Supplementary Figure 1

Simul-seq library preparation and quality control.

(**a**) Histogram of incubation times for parallel Tru-seq DNA and RNA library preparation as well as Simul-seq. (**b**) High-sensitivity DNA bioanalyzer trace for a yeast/human mixed Simul-seq library. Note, this trace is representative of an average Simul-seq library. (**c-d**) Representative droplet digital PCR (ddPCR) raw fluorescence amplitude data (**left**) and assay design (**right**) for quantification of DNA (**c**) and RNA (**d**) constituents of Simul-seq libraries.

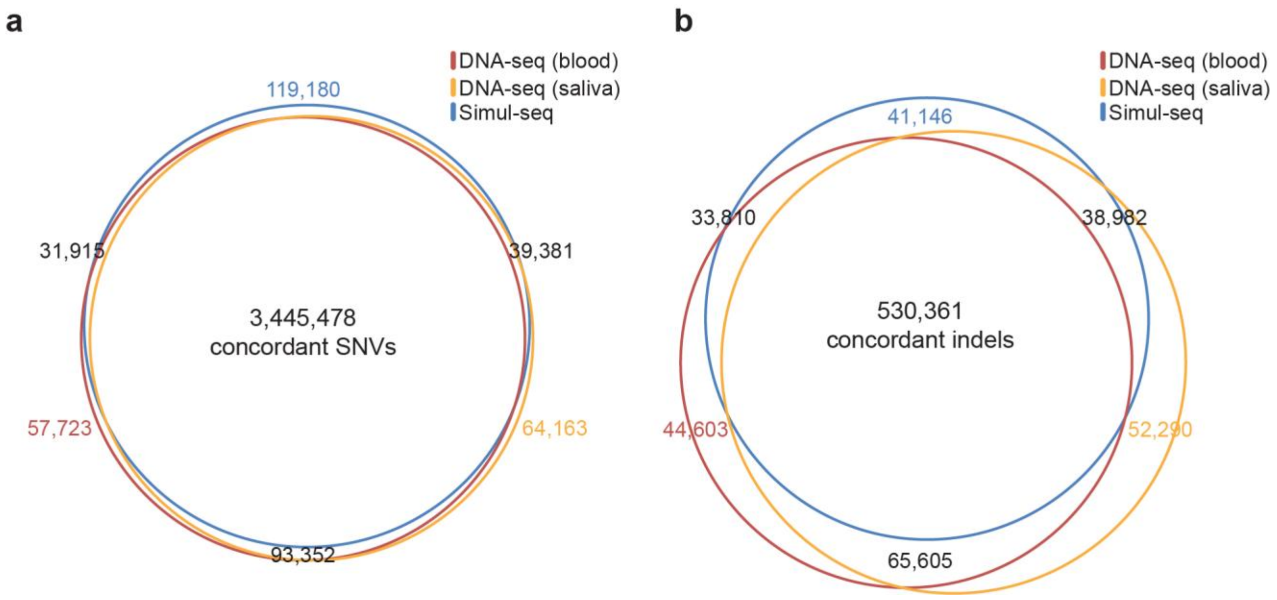

Supplementary Figure 2

Comparison of variant calls between Simul-seq genome and DNA-seq replicates.

(**a-b**) Venn diagrams comparing SNV (**a**) and indel (**b**) calls between the Simul-seq genome and two DNA-seq control genomes derived from different tissues of the same individual[13].

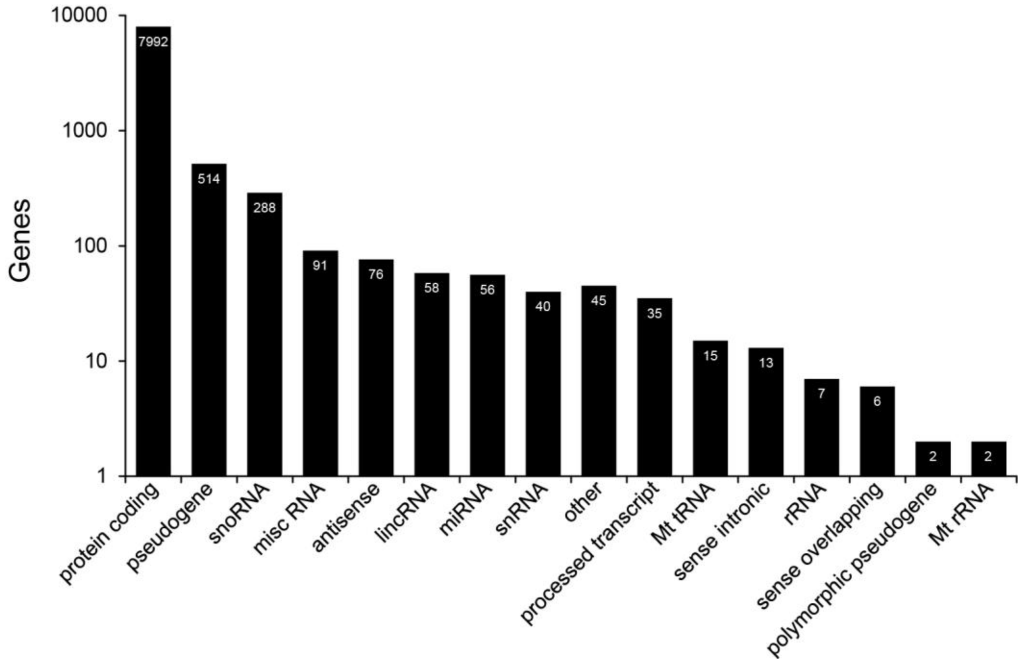

Supplementary Figure 3

Distribution of coding and noncoding genes in Simul-seq transcriptome.

Bar graph of all Ensembl biotype annotations for genes with FPKM values greater than or equal to 5.

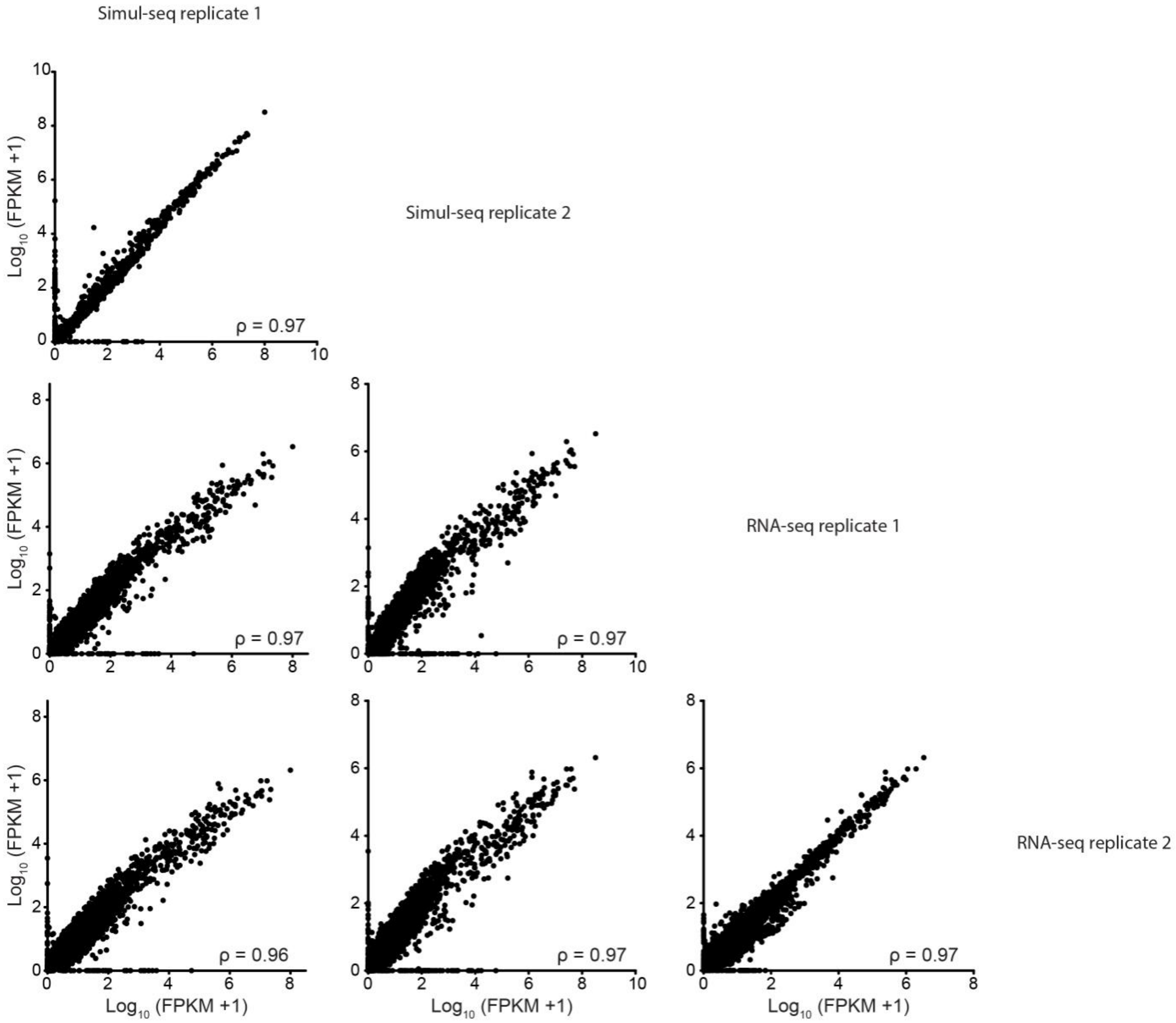

Supplementary Figure 4

Simul-seq and RNA-seq replicates are well correlated.

Scatter plots of Log_{10}(FPKM+1) gene measurements for Simul-seq and RNA-seq replicates. Spearman's ρ correlation values for each comparison are shown.

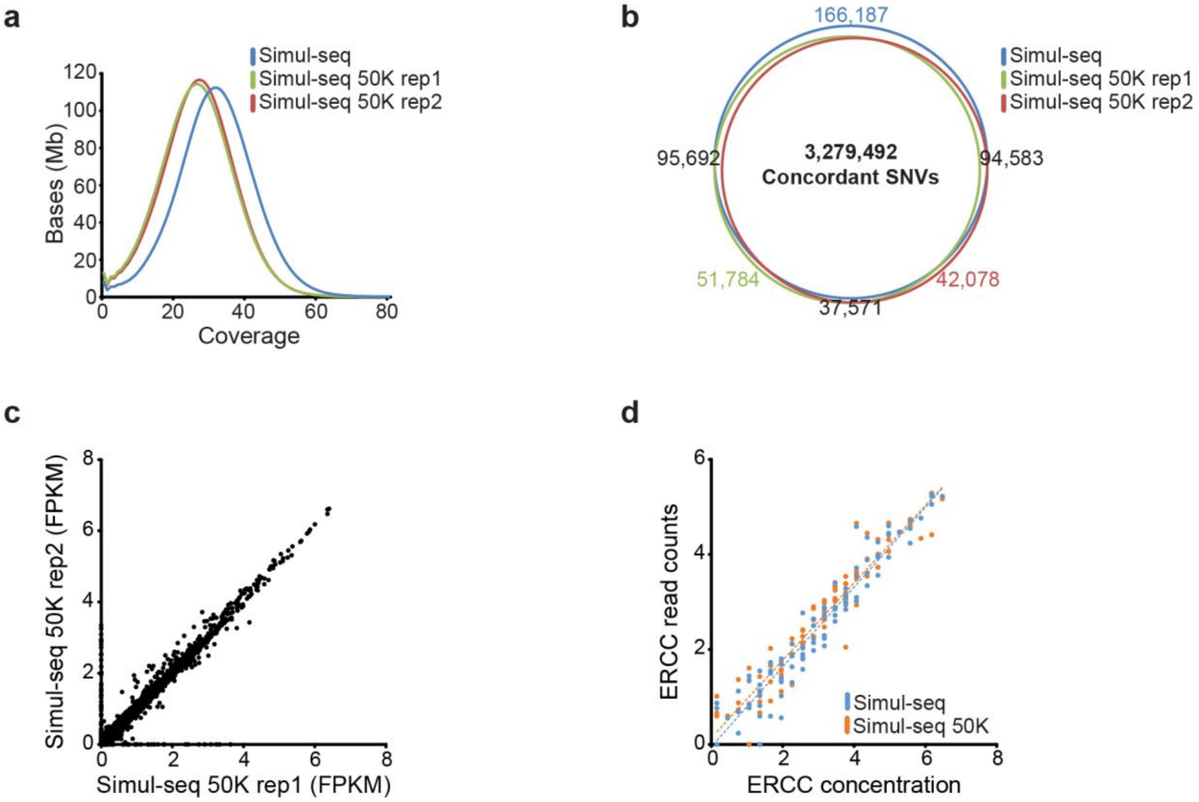

Supplementary Figure 5

DNA and RNA sequencing data for 50,000 (50K) fibroblast replicates.

(**a**) Coverage distributions for Simul-seq libraries of the same individual. (**b**) Venn diagrams comparing SNV calls between the Simul-seq replicates. (**c**) Scatter plots of Log_{10}(FPKM+1) gene measurements for 50K Simul-seq replicates (Spearman's ρ=0.97). (**d**) Correlation between External RNA Controls Consortium (ERCC) spike-in control Log_{10} RNA concentrations versus the average Log_{10}(RPKM+1) for Simul-seq (blue; Spearman's ρ=0.97) and 50K Simul-seq (orange; Spearman's ρ=0.96) fibroblast replicates (n=2/group). Note, zero values have been shifted to 1, and all ERCC transcripts are shown.

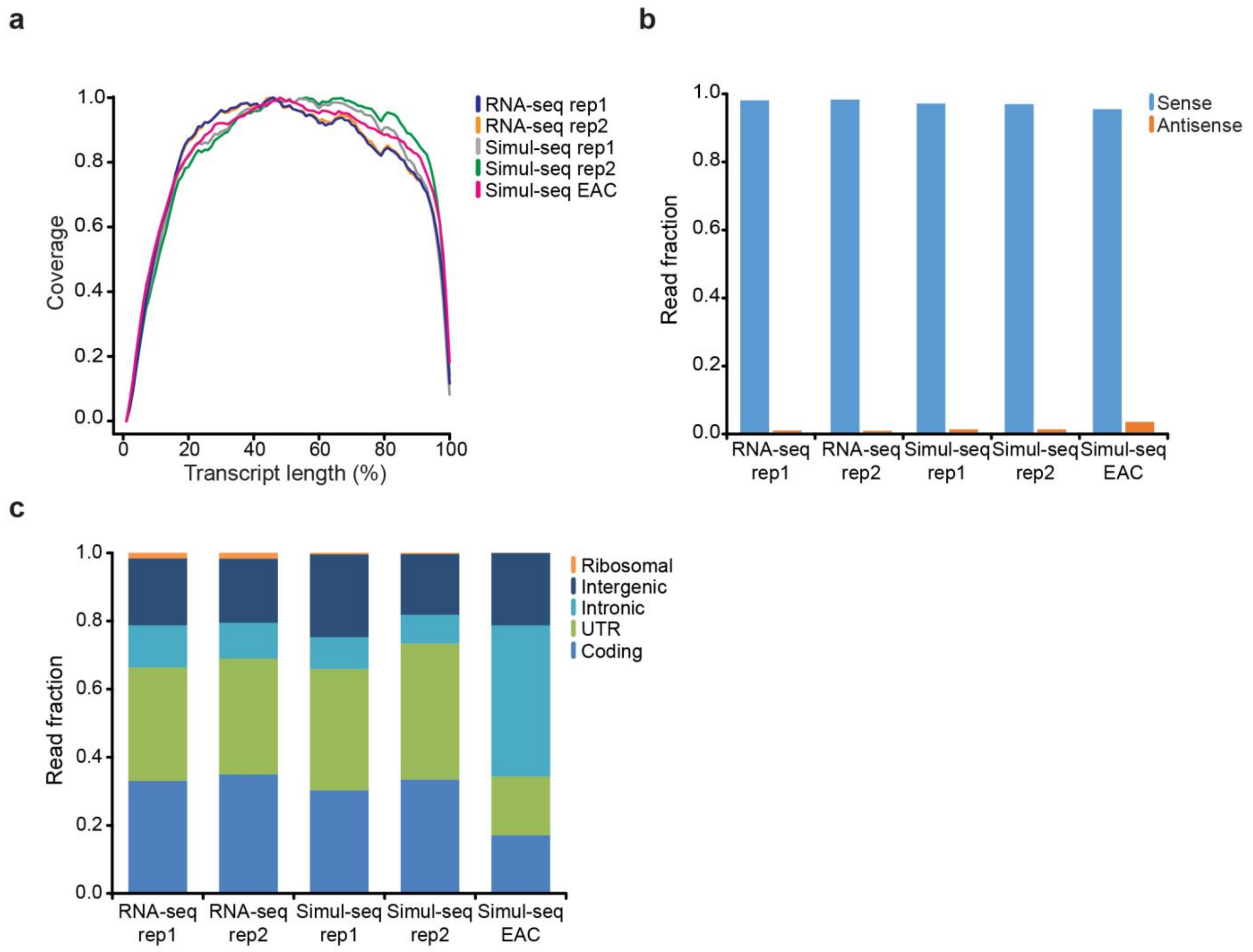

Supplementary Figure 6

Simul-seq replicate and tumor RNA quality control analysis.

(**a**) Distribution of normalized transcript coverage for RNA-seq and Simul-seq replicates performed on fibroblasts as well as Simul-seq data obtained for esophageal adenocarcinoma tissue isolated using laser capture microscopy (Simul-seq EAC). (**b**) Strand specificity of Simul-seq and RNA-seq samples. (**c**) The fraction of reads mapping to various genomic annotations for Simul-seq and RNA-seq samples. Note, an increased intronic read fraction combined with a similar intergenic read fraction in the Simul-seq EAC sample likely indicates increased intron retention and/or a higher proportion of unspliced RNA in this specimen.

Nature Methods: doi:10.1038/nmeth.4028

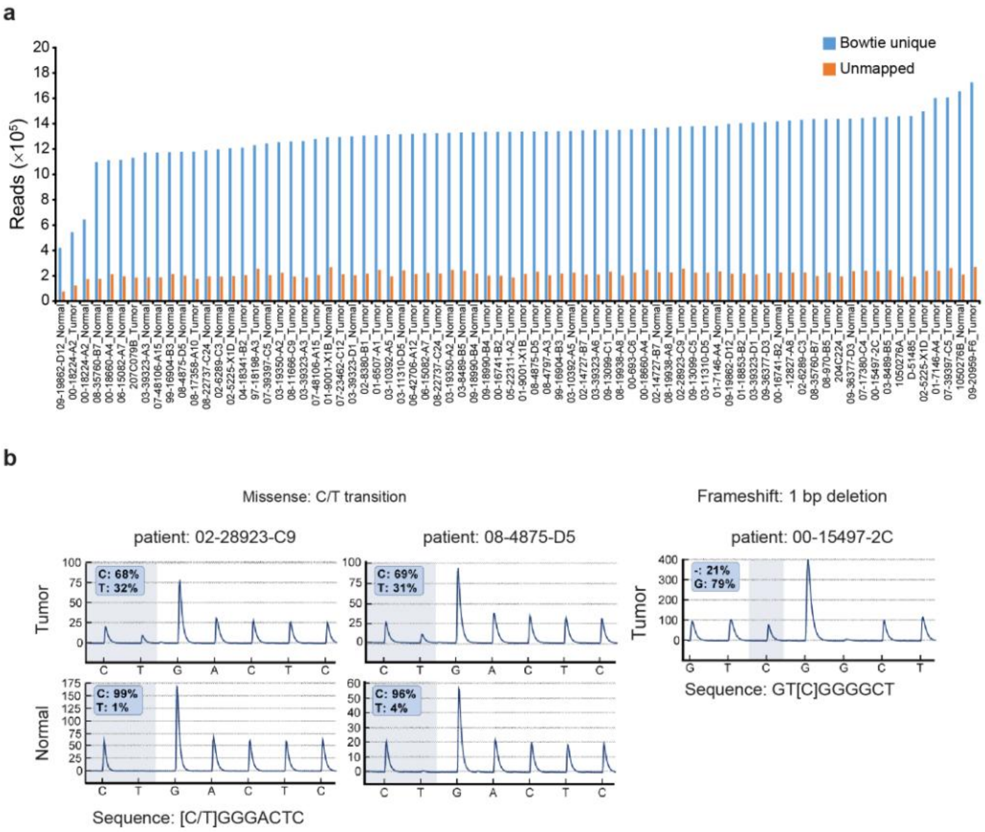

Supplementary Figure 7

Targeted resequencing of *KIF3B* locus in esophageal adenocarcinoma patient samples.

(**a**) Histogram of the unique and unmapped Bowtie aligned reads obtained for 76 FFPE samples (50 tumors and 26 normals). The original sample (02-28923-C9) that was subjected to the Simul-seq protocol was included as a positive control. A single tumor-normal pair (00-18224-A2) displayed a substantially higher number of variant calls yet a lower number of uniquely mapped reads, suggesting that these samples harbored increased rates of PCR errors induced by low quality genomic DNA. Therefore, these samples were not included in somatic mutation analysis. (**b**) Validation of variant calls using pyrophosphate sequencing.

Nature Methods: doi:10.1038/nmeth.4028

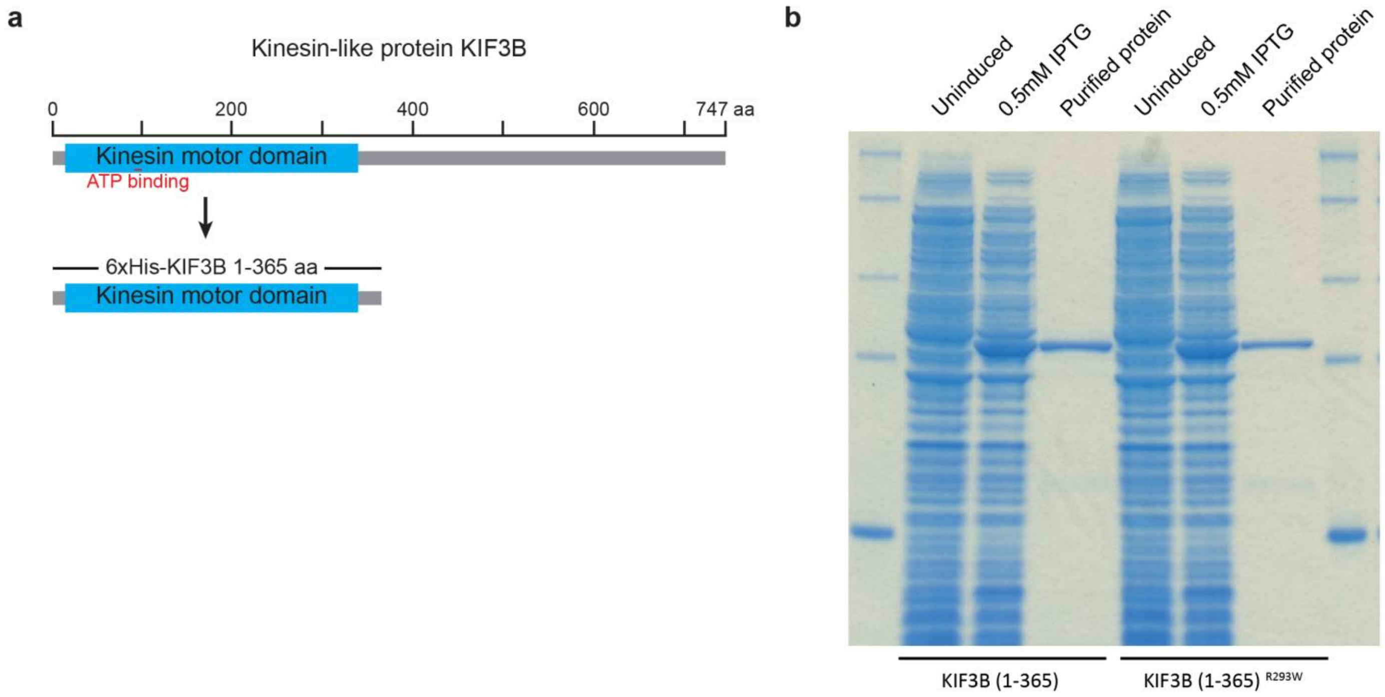

Supplementary Figure 8

Purification of recombinant wild-type and R293W mutant motor domains.

(**a**) Schematic of KIF3B protein, with motor domain and ATP binding region highlighted in blue and red, respectively. For biochemical assays, a region spanning the motor domain of KIF3B (amino acids 1-365) was cloned and recombinantly expressed with an N-terminal 6x-Histidine tag (**bottom**). (**b**) Coomassie stained gel of recombinant proteins pre- and post-induction with Isopropyl β-D-1-thiogalactopyranoside (IPTG) as well as after Ni^{2+} affinity purification.

Supplementary Information

Comprehensive genome and transcriptome profiling using simultaneous DNA and RNA sequencing (Simul-seq)

Jason A. Reuter[1,4], Damek V. Spacek[1,4], Reetesh K. Pai[2,3], and Michael P. Snyder[1]

[1] Department of Genetics, Stanford University School of Medicine, Stanford, CA, USA

[2] Department of Pathology, Stanford University School of Medicine, Stanford, CA, USA

[3] Present address: Department of Pathology, the University of Pittsburgh Medical Center, Pittsburgh, PA, USA

[4] These authors contributed equally to this work.

Correspondence should be addressed to M.P.S. (mpsnyder@stanford.edu)

Supplementary Note:

Detailed description of Simul-seq reagents, equipment and protocol

Reagents
0.2 mL PCR tubes
Molecular Probes Qubit assay tubes: Q32856
1.5 mL Eppendorf DNA LoBind microcentrifuge tubes: 022431021
Qiagen RNeasy mini: 74104
Qubit dsDNA HS kit: Q32851
Qubit RNA HS kit: Q32852
Illumina RiboZero Magnetic Gold: MRZG126
Zymo Research RNA Clean and Concentrator 5 kit: R1015
NEB Small RNA Library Preparation Kit: E7330S
Illumina Nextera Library Preparation Kit: FC-121-1031
Illumina Nextera Index Kit: FC-121-1012
NEB RNase III: E6146S
Beckman Coulter Ampure XP RNAclean beads: A63987
Beckman Coulter Ampure XP beads: A63880
HPLC-purified custom NEB PCR primers (*denotes phosphorothioate bond)(all 100 nmole from IDT):
NEB SR 503
5′-AATGATACGGCGACCACCGAGATCTACACTATCCTCTGTTCAGAGTTCTACAGTCCG*A-3′
NEB SR 505
5′-AATGATACGGCGACCACCGAGATCTACACGTAAGGAGGTTCAGAGTTCTACAGTCCG*A-3′
NEB 702
5′-CAAGCAGAAGACGGCATACGAGATCTAGTACGGTGACTGGAGTTCAGACGTGTGCTCTTCCGATC*T-3′
NEB 703
5′-CAAGCAGAAGACGGCATACGAGATTTCTGCCTGTGACTGGAGTTCAGACGTGTGCTCTTCCGATC*T-3′
(Optional reagents)
IDT PrimeTime Std qPCR Assays for custom ddPCR-based library quantification:
  DNA forward primer (9.0 nM): 5′-AAGCAGAAGACGGCATACGAG-3′
  DNA reverse primer (9.0 nM): 5′-GCGACCACCGAGATCTACAC-3′
  DNA 5′ probe (2.5 nM; HPLC-purified): 5′-CTTATACACATCTGACGCTGCCGACG-3′ with 5′-6-FAM fluorophore and internal ZEN and 3′-Iowa Black FQ dual quenchers
  DNA 3′ probe (2.5 nM; HPLC-purified): 5′-TCTCTTATACACATCTCCGAGCCCACG-3′ with 5′-HEX fluorophore and internal ZEN and 3′-Iowa Black FQ dual quenchers
  RNA forward primer (9.0 nM): 5′-AAGAGAAGACGGCATACGAG-3′
  RNA reverse primer (9.0 nM): 5′-GCGACCACCGAGATCTACAC-3′
  RNA 5′ probe (2.5 nM; HPLC-purified): 5′-CTGTTCAGAGTTCTACAGTCCGACGATC-3′ with 5′-6-FAM fluorophore and internal ZEN and 3′-Iowa Black FQ dual quenchers
  RNA 3′ probe (2.5 nM; HPLC-purified): 5′-ACTGGAGTTCAGACGTGTGCTCTTCC-3′ with 5′-HEX fluorophore and internal ZEN and 3′-Iowa Black FQ dual quenchers
Bio-Rad 2× digital droplet PCR supermix for probes (No dUTP): 186-3023

Bio-Rad droplet generation oil for probes: 1863005
Bio-Rad DG8 Cartridges: 1864008
Bio-Rad DG8 Gaskets: 1863009
Bio-Rad ddPCR Droplet Reader Oil: 1863004
Eppendorf 96-well PCR plate: 951020362

Equipment
Microcentrifuge (general lab supplier)
Qubit Fluorometer (Life Technologies)
Magnetic stand for 1.5 ml tubes (general lab supplier)
Thermal cycler with programmable lid temperature (general lab supplier)
Heat block (general lab supplier)
QX200 ddPCR system, with PX1 PCR Plate Sealer (Bio-Rad) (*optional equipment*)

Procedure

A) Nucleic acid isolation:

1. Extract total nucleic acids from cell or tissue samples using the Qiagen RNeasy mini kit per manufacturer's instructions, except without the optional DNase I treatment. Elute total nucleic acids in 30 µl of molecular biology grade, nuclease free H_20 (henceforth called H_20).
2. Use dsDNA Qubit to quantify genomic DNA concentration and RNA Qubit to quantify total RNA concentration for subsequent RiboZero depletion.

B) RiboZero magnetic gold ribosomal RNA depletion:

1. Starting with between 0.5-5 µg of total RNA as measured with the RNA Qubit (with concomitant genomic DNA present), use the Illumina RiboZero Magnetic Gold Depletion kit per manufacturer's instructions with the following change, for step 2.3 change temperature of incubation to 65 °C from 68 °C.
2. Use the Zymo Research RNA Clean and Concentrator 5 cleanup kit following the manufacturer's protocol to recover all nucleic acids >17 nucleotides and elute in 12 µl of H_20.
3. Use dsDNA Qubit to quantify genomic DNA (gDNA) concentration and RNA Qubit to quantify RNA concentration for tagmentation/fragmentation reaction.

Protocol can be safely paused overnight here if necessary. Store samples at -80 °C.

C) Simultaneous tagmentation of gDNA and fragmentation of RNA:

1. Set up the following reaction with 50 ng of genomic DNA and the corresponding amount of ribosomally depleted RNA in a 0.2 mL PCR tube. Between 5 -100 ng of starting RNA should be present per 50 ng of gDNA.
    a. 50 ng gDNA
    b. 25 µl TD buffer (Nextera kit)
    c. 5 µl TDE (Nextera kit)
    d. Y µl H20 (50 µl total reaction volume)
    e. 1 µl RNase III (1:1 dilution in TD buffer; 0.5 units of RNase III)
2. Mix well (50 µl) and use a thermal cycler to incubate at 55 °C for 5 minutes and cool to 10 °C; immediately place on ice and proceed to next step.

D) Ampure cleanup of tagmentation/fragmentation reaction:

1. Transfer sample to 1.5 mL Eppendorf tube and add 100 µl (or 2× of reaction volume) of Ampure XP RNA clean beads to the 50 µl reaction.
2. Incubate for 10-15 minutes at room temperature and then place on magnetic stand.
3. Wash 2× with 400 µl of 80% ethanol.
4. Air dry for 10 minutes and resuspend in 7 µl of H_20.

E) Removal of RNA secondary structure:

1. Transfer 6 µl of the elution from the tube on the magnetic stand to a new 0.2 mL PCR tube.
2. Add 1 µl of 3′ adapter (NEB small RNA kit).
3. Incubate in preheated thermal cycler for 2 minutes at 65 °C and immediately place on ice.

F) 3′ adapter ligation:

1. Set up the following reaction on ice to ligate the 3′ adapter onto the RNA, using reagents from the NEB small RNA library preparation kit.
    a. 10 µl 3′ Ligation Buffer (2×)
    b. 3 µl 3′ Ligation Enzyme Mix
2. On ice add 13 µl of 3′ ligation master mix to the 7 µl of denatured gDNA/RNA.
3. Mix well (20 µl) and incubate for 1 hour at 25 °C in thermal cycler, with lid heated to 55 °C; hold at 4 °C.

G) Hybridization of reverse transcription oligo:

1. Set up the following reaction on ice to hybridize the RT oligo to the 3′ adapter, using reagents from the NEB small RNA library preparation kit.
    a. 4.5 µl H_20
    b. 1 µl SR RT Primer
2. Add 5.5 µl of diluted RT primer to the 20µl 3′ ligation reaction.
3. Mix well (25.5 µl) and use a thermal cycler to incubate the samples for 5 minutes at 65 °C, 15 minutes at 37 °C and then 15 minutes at 25 °C; hold at 4 °C.
4. With ~5 minutes remaining, prepare the 5′ SR adaptor by resuspending in 120 µl of H_20.
5. Aliquot 1.1× of resuspended 5′ SR adaptor and denature for 2 minutes at 70 °C; immediately place on ice. Store excess adapter in aliquots at -80 °C for subsequent use.

H) Ligate 5′ SR adaptor:

1. Set up the following reaction on ice to ligate the 5′ adaptor onto the RNA, using reagents from the NEB small RNA library preparation kit.
    a. 1 µl 5′ SR adaptor
    b. 1 µl 5′ Ligation Reaction Buffer
    c. 2.5 µl 5′ Ligase Enzyme Mix
2. Add 4.5 µl of the 5′ ligation master mix to the 25.5 µl RT hybridization reaction.
3. Mix well (30 µl) and incubate for 1 hour at 25 °C, with lid heated to 55 °C; hold at 4 °C.

I) cDNA synthesis:

1. Set up the following reaction on ice to make first strand cDNA master mix, using reagents from the NEB small RNA library preparation kit.
    a. 8 µl First Strand Reaction Buffer (5×)
    b. 1 µl Murine RNase Inhibitor
    c. 1 µl ProtoScript II Reverse Transcriptase
2. Transfer 30 µl of 5′ ligation reaction to a new 0.2 mL PCR tube and add 10 µl of cDNA synthesis master mix.

3. Mix well (40 µl) and use a thermal cycler to incubate for 1 hour at 50 °C, with lid heated to 55 °C; hold at 4 °C.

J) Ampure cleanup of gDNA and cDNA synthesis reaction:

1. Transfer sample to new 1.5 mL Eppendorf and add 1.2× Ampure XP beads.
2. Incubate for 5-10 minutes at room temperature; place on magnet.
3. Wash 2× with 400 µl of 80% ethanol.
4. Air dry for 5-10 minutes and resuspend in 26.5 µl of H_20; place on magnet.
5. Transfer 25.5 µl of elution to a new 0.2 mL PCR tube.

Protocol can be safely paused overnight here if necessary. Store sample at -20 °C.

K) gDNA/cDNA library PCR amplification:

1. Set up the following PCR reaction to enrich for the cDNA, using PCR reagents from the Nextera library preparation kit and custom multiplex primers (NEB 70X and NEB SR 50X for forward and reverse, respectively). More guidelines for cycle number can be found in the Critical Steps section below.
    a. 25.5 µl gDNA/cDNA library mixture
    b. 1.25 µl custom NEB SR 50X (10 µM stock)
    c. 1.25 µl custom NEB 70X (10 µM stock)
    d. 12 µl NPM
2. Mix well (40 µl) and perform thermocycling as follows for 2-7 cycles, depending on RNA input.
    a. 72 °C, 3 minutes
    b. 98 °C, 30 seconds
    Repeat following cycle 2-7 times
    c. 98 °C, 10 seconds
    d. 62 °C, 30 seconds
    e. 72 °C, 3 minutes
    f. Hold at 4 °C
3. Once the PCR reaction is at 4 °C, add the PCR master mix below to amplify the final gDNA/cDNA libraries, using PCR reagents and primers from the Nextera library preparation kit.
    a. 40 µl gDNA/cDNA library
    b. 2.5 µl N70X (5 µM stock)
    c. 2.5 µl N50X (5 µM stock)
    d. 5 µl PPC
    e. 5 µl NPM
4. Mix well (55 µl) and perform thermocycling as follows for 5 cycles
    a. 72 °C, 3 minutes
    b. 98 °C, 30 seconds
    Repeat following cycle 5 times
    c. 98 °C, 10 seconds
    d. 62 °C, 30 seconds
    e. 72 °C, 3 minutes
    f. Hold at 4 °C

L) Ampure cleanup of final library:

1. Transfer sample to new 1.5 mL Eppendorf and add 1.2× Ampure XP beads to 55 µl reaction.

2. Incubate for 5-10 minutes at room temperature and place on magnet.
3. Wash 2× with 400 µl of 80% ethanol.
4. Air dry for 5-10 minutes and resuspend in 12.5 µl of H_20; place on magnet.
5. Transfer 12 µl of elution to a new 1.5 mL Eppendorf tube.
6. Use Qubit dsDNA HS to quantify final library concentration. Agilent Bioanlyzer High Sensitivity kit can be used to visualize the library size. A typical Simul-seq library will be approximately 10 ng/µl, with an average size distribution of ~350 bp (**Supplementary Fig. 1b**).

M) (OPTIONAL) ddPCR quantification of libraries

1. ddPCR experiments are performed according to manufacturer's guidelines, using a Bio-Rad QX200 system.
2. Equally mix 5′ and 3′ IDT PrimeTime Std qPCR assays for ddPCR-based library quantification of either the RNA (NEB) or the DNA (Nextera) constituents of the Simul-seq library. Note, assaying both ends ensures that both 5′ and 3′ adapters are correct.
3. Assemble triplicate 20 µl ddPCR reactions at room temperature:
    a. 10 µl of ddPCR 2× super mix (Bio-Rad)
    b. 1 µl of combined NEB or Nextera ddPCR assay mixes
    c. 2 µl of diluted Simul-seq libraries (10^{-6} dilution often is sufficient but will vary depending on the starting library concentration).
    d. 7 µl of H_20
4. Generate droplets by adding the 20 µl ddPCR reactions to the sample wells of a DG8 cartridge (Bio-Rad) and then adding 70 µl of droplet generation oil for probes (Bio-Rad) to oil wells; place a DG8 gasket (Bio-Rad) over the cartridge and insert into the QX200 droplet generator. Transfer droplets into an Eppendorf 96-well PCR plate and repeat until droplets have been produced for all of the ddPCR reactions.
5. Cover PCR plate with foil using the plate sealer and subject the ddPCR reactions to the following PCR cycling program:
    a. 10 minutes at 95 °C

    40 cycles of

    b. 30 seconds 95 °C
    c. 1 minute at 60 °C
    d. Followed by 10 minutes at 98 °C
    e. Hold at 4 °C
6. Transfer plate to the QX200 droplet reader and quantitate library ratios with QuantaSoft software (**Supplementary Fig. 2c,d**).

Critical Steps

Use proper RNA handling procedures throughout as RNases can lead to degradation and lower quality libraries.

Step B-3: Measurement of the gDNA prior to tagmentation is critical because 50 ng should be added to the reaction for optimal results.

Step C-1: The tagmentation/fragmentation step needs to be carried out without interruption as prolonged RNase III activity may over fragment the RNA.

Step D-1: Ampure XP RNA clean up beads should be used in this step, as column-based purification appeared increased bias in Simul-seq DNA reads.

Step D-2: Full incubation time for the Ampure XP RNA cleanup is critical to maximize recovery of both DNA and RNA.

Step K-1: Choosing the correct number of cycles for the cDNA enrichment PCR is important for an optimal ratio of DNA/RNA reads in the final library (**Fig. 1c**). General guidelines based on the starting amount of ribosomally depleted RNA are as follows: 7 cycles for 5-15 ng, 5 cycles for 15-30 ng, 3 cycles for 30-50 ng, 2 cycles for >50 ng. Variability between sources of starting material may affect final library ratios. It is recommended that the user tests the number of PCR cycles when changing a source of starting material.

Step K-2: For the final library amplification PCR, 5 cycles worked well for all experiments that were undertaken.

Step M: DNA:RNA ratios of between 5-10:1 are optimal for whole genome and whole transcriptome sequencing of human samples. ddPCR ratios are well correlated to final read counts (**Fig. 1d**) if alternative ratios are desired.

Sequencing must be performed with Illumina dual index reads to ensure proper adapter pairing. NEB PCR primers have been remade with normal Nextera indices for ease of pooling (see reagents). Nextera low plex pooling guidelines should be followed to balance index reads, allowing for optimum library yields. Finally, this library design is only compatible with MiSeq and HiSeq Illumina platforms.
